# Expert insights on digital contact tracing: interviews with contact tracing policy professionals in New Zealand

**DOI:** 10.1093/heapro/daac059

**Published:** 2022-07-05

**Authors:** Tim Chambers, Richard Egan, Karyn Maclennan, Tepora Emery, Sarah Derrett

**Affiliations:** Department of Public Health, University of Otago, Wellington, New Zealand; Department of Preventive & Social Medicine, University of Otago, Dunedin, New Zealand; Ngāi Tahu Māori Health Research Unit, Department of Preventive & Social Medicine, University of Otago, Dunedin, New Zealand; Toi-Ohomai Institute of Technology, Rotorua, New Zealand; Department of Preventive & Social Medicine, University of Otago, Dunedin, New Zealand

**Keywords:** COVID-19, digital contact tracing, policy, public health

## Abstract

Digital contact tracing (DCT) is the application of digital tools to assist with identifying and informing close contacts of a COVID-19 case. DCT is a potential solution to capacity constraints of current manual contact tracing processes. Expert opinion from contact tracing professionals rarely informs public discourse on the benefits and limitations of DCT solutions. Three focus groups were undertaken in New Zealand to understand benefits and limitations of DCT solutions from contact tracing professionals. One was with the National Investigation and Tracing Centre (NITC) and two were with Public Health Units (PHUs). Participants highlighted four key themes including: (i) equity, (ii) privacy, (iii) communication and public perception and (iv) the operational model. Participants were concerned DCT solutions could exacerbate existing health inequities due to lack of access to, or familiarity with, technology. Poor communication and public understanding of DCT were seen as a major threat to both the efficacy of DCT solutions and the wider COVID-19 response. Most importantly, end-users were cautious of the operational model for DCT data that might: (i) attempt to replace manual processes that cannot or should not be automated by technology (case investigations, follow-ups); (ii) place undue burden on citizens and (iii) increase the workload for the current system beyond its capacity, for unproven or limited benefit. To be effective, contact tracing professionals believed DCT technologies must have strong privacy safeguards, a clear and simple communication strategy, interoperability with the existing contact tracing system and a foundation of health equity.

## BACKGROUND

The global COVID-19 pandemic highlights the importance of accurate and timely contact tracing. Digital contact tracing (DCT) is a potential solution to capacity constraints of current manual tracing processes (Von Wyl *et al.*, 2020). Modelling studies have demonstrated reductions in secondary cases if DCT solutions are used together with other public health measures (Ferretti *et al.*, 2020; Hinch *et al.*, 2020; Kucharski *et al.*, 2020; Plank *et al.*, 2022). However, there remains limited empirical evidence of the efficacy of DCT technologies (Anglemyer *et al.*, 2020). Despite the lack of empirical evidence, most countries, including New Zealand, have embarked on and implemented some form of DCT technology.

The focus of DCT technology research has been on modelling its effectiveness (hypothetically, could it help?) (Ferretti *et al.*, 2020; Hinch *et al.*, 2020; Kucharski *et al.*, 2020; Plank *et al.*, 2022); public acceptability (will the public accept its implementation?) (Altmann *et al.*, 2020; Colmar Brunton, 2020); privacy and legal issues (is it lawful? and does it impact rights to privacy?) (Von Wyl *et al.*, 2020) and equity focus (how will its implementation increase or decrease health inequities?) (Von Wyl *et al.*, 2020). However, more practical issues of technology interoperability with existing contact tracing infrastructure, the public health capacity to implement and use data, and the potential adverse public health impact of these technologies are largely absent. There are calls for the wider issues of DCT such as de-centralization of public health and corporate influence to be discussed (French *et al.*, 2020). End-users, that is contact tracers and contact tracing policy professionals, are likely to provide the expert knowledge to better understand the operational constraints and ethical challenges of DCT technologies from a public health perspective. However, the views of these health professionals in epidemic response situations are rarely represented (Walker *et al.*, 2020).

In New Zealand, multiple reports highlighted systemic problems in the contact tracing systems that led to inefficiencies and health inequities (Allen and Clarke, 2020; Contact Tracing Assurance Committee, 2020; Verrall, 2020). DCT was proposed to help contact tracers improve the speed and accuracy of contact tracing; some DCT solutions were designed with the specific intention to reduce existing health inequities. In response, in mid-2020, the New Zealand Ministry of Health (MoH) established a Contact Tracing Technologies Research Programme (‘the Programme’). The Programme included research around a Bluetooth contact tracing card (‘Card’). The card did not contain location-based technology, such as GPS, so user locations were not tracked. Algorithms on the card assessed the radio signal strength indicator (RSSI) over the duration of an encounter to identify close contact interactions. A full technical assessment of the Card was completed by the New Zealand Defence Technology Agency and is publicly available (De Lautour *et al.*, 2020).

The Programme included: (i) a public field trial of the Card in a community in Rotorua; (ii) a technical trial of the Card in a controlled setting; (iii) consumer research on the public acceptability of the Card; (iv) privacy impact assessments; (v) equity reports and (vi) focus groups with contact tracing professionals. This research reports on three focus groups with end-users on the potential for the Bluetooth card and phone-based applications (from here on simply referred to as ‘DCT technologies’) to facilitate contact tracing processes. The research aimed to understand how contact tracers and contact tracing policy professionals would use digital contact data (i.e. COVID phone App or the Card being tested) within the existing contact tracing infrastructure and consider how effective these technologies might be at capturing contacts and enhancing contact tracing.

## METHODS

This research adopts a pragmatic interpretive framework. Pragmatic frameworks are beneficial when the focus of inquiry is ‘what works?’ and ‘how does it work?’(Creswell and Poth, 2016). Pragmatic approaches are useful when researchers are interested in understanding an issue (in this case, DCT technologies) and learning about their potential strengths and obstacles.

Primary qualitative data were collected in Wellington, Auckland and Palmerston North, New Zealand. Three focus groups were conducted. The first focus group was face-to-face with the National Investigation and Tracing Centre (NITC), based within the MoH in Wellington on 14 October 2020. The NITC was established in 2020 to support case investigation and close contact tracing to manage and monitor infectious notifiable diseases (Ministry of Health, 2020a). The NITC is responsible for ensuring providers and other staff have access to the National Contact Tracing Solution (NCTS), an IT system that supports the end-to-end contact tracing process. When required, the NITC ensures that close contacts of COVID-19 cases are called within 48 h of registration by the Public Health Units (PHUs) by operating through call center providers.

The second and third focus groups were with case investigators from two different PHUs. The 12 PHUs across New Zealand are responsible for delivering public health services with a focus on environmental health, communicable disease control, tobacco control and health promotion programmes. PHUs conduct the initial case investigation of an index case, which identifies close and casual contacts (Ministry of Health, 2020a). PHUs also provide health and social support for the case and follow-up services. One focus group was face-to-face on 15 October 2020 and the other was via Zoom on 2 December 2020.

All focus groups were held at the participants’ workplaces, with lunch provided and a general sense of informal professionalism. All participants received the information sheet and consent form before the focus group, and were reminded in person about confidentiality issues. Conducted over 60–90 min, each focus group began with *whakawhanaungatanga/introductions*. The interview guide was designed to allow participants to provide their expert opinion on DCT technologies in general. Two examples were used throughout, a hardware-based Card solution (‘Card’) and a software-based phone solution (an app).

Focus group discussions were audio-recorded for thematic and interpretive analysis (Wolcott, 1994; Braun and Clarke, 2006). Field notes were written up after the focus groups, including preliminary reflections about emerging themes. A mix of NVivo software and marking up Microsoft Word transcripts was used to analyse the audio-recordings and the field notes. Coding was done independently by RE and TC, then combined for coherence. RE had 21 codes, TC had eight codes, of which three were the same as RE. Upon further discussion, including with the wider research team, these codes were collapsed into four distinct themes. Memos, reflecting on the transcription, were written during the analysis. The wider team reviewed the draft thematic analysis.

## RESULTS

Table 1 shows participants’ demographics. The majority of participants were aged over 45 years. Job roles in the NITC were a mix of operational, clinical and managerial positions. PHUs included public health nurses, health protection officers, contact tracing team leads, communicable disease nurses and an administrative technician.

**Table 1:** Participant demographic characteristics

	Total
Total	23
Ethnicity	
NZE	18
Māori	2
Other	3
Sex	
Male	7
Female	16
Age	
25–34	5
35–44	3
45–54	8
55–64	7

Four key themes were identified: equity, communication and public perceptions, privacy and operational model. Each theme is explained and supported with indicative quotes.

### Equity

Equity is central to public health work and was important for participants. This was succinctly expressed by a NITC participant,

If we’re not using the technology to improve equitable outcomes, should we be using it at all? A colleague has described the first outbreak … affecting the traveling well [i.e. people who could afford to travel], and actually the second current outbreak … largely Pacific populations. We know that as a virus, COVID-19 will find vulnerability within society. So actually, it [equity] has to be front and centre of any consideration around development of tools. How much does this cost, and is this more beneficial for equitable outcomes as opposed to using that money to do something that responds directly to the needs of the most vulnerable communities? So, I think the trade-off of resources is top of mind (NITC).

The PHU focus groups were also cognisant of equity and diversity issues, raising concerns about access and efficacy for marginalized populations,

I have a concern about an assumption that … that everyone has a cell phone. Because there’s a huge population of the very young and the very old that don’t. Those two groups may be the most vulnerable (PHU).

There’re the poor people who can’t afford it [phone], the older generation don’t know how to work them, … [and] It’s not an easy thing to actually be able to download and to be able to use [the COVID App] (PHU).

Similarly, there was concern about availability of technology, a barrier that was noted from PHU staff,

Some of those families especially in South Auckland with a high population of Pacific people there, there’s only one phone in the family. If you have 10 people in those families and Dad only has the phone that’s for everybody. … The one person who had that phone will get a ping from the Card or from another App, and it pays to ask that person is there anybody else with them at that moment when they were pinged. … That was what we learned up there from some of the families (PHU).

A possible solution to this issue was a suggestion, ‘What about if you just gave people phones’ (PHU).

### Privacy

Privacy concerns were clearly an issue and a challenge across the focus groups. The current system does not allow for full investigation due to privacy issues,

We have no ability because of privacy to pull [individual data] and know who the individuals are, who they have been at the event with, you can push the notification [asking people to make contact with the contact tracers] but you can’t pull. So, in some ways, we have lost the ability to know who those individuals are to get in contact with them (NITC).

Further, indicatively it was suggested, ‘it [new technology such as the Card] has to have very strong privacy controls around it’ (NITC focus group); and similarly,

I think one of the questions that often comes up when you are talking about this, what exactly are you going to be collecting? A lot of people are scared about what information is going to be collected about them and then what is going to happen to that information (PHU).

The contact tracing systems currently used do not allow for centralized information, therefore linking contacts is more difficult. Increased capacity to electronically link data for tracing was discussed, with a NITC participant noting, ‘It has been raised and recognised that [it would] take a change of legislation, which is impossible now but might be in the coming times’ (NITC). A central issue was the privacy versus efficacy challenge.

In one PHU, it was also recognized that their staff needed to be clear about how the technologies worked, ‘Some of it is about us having a good understanding as well, about how that technology works’ (PHU). Privacy was therefore connected to the communications issues presented below, with simplicity and clarity needed to ensure PHU staff, and the public, understand both the technology and how the data will be used and accessed.

### Communication and public perceptions

Visibility, simplicity and some frustrations were at the forefront of participants’ concerns about how the public understand and hear messages about COVID-19. For example, ‘making sure that there is good visibility of contact tracing in the work that we do’ was important to the NITC group. There was a clear focus on ‘how people will receive this [digital contact tracing technology]?’ (PHU), with all participants cognisant of public perceptions of Government overreach and surveillance.

Focus group participants were very aware of the importance of having the public informed, on board and actively participating in the collective COVID-19 response. The current COVID App was seen as useful, and even with variable use, simple to use. The limitations regarding cell phone ownership, coverage, disability, etc. were noted, therefore there was an openness to improvements from the status quo. In fact, it was noted the COVID App was not being fully utilized, ‘Even though the App is doing a lot of good things, in some ways it’s not fully enhanced the process and created difficulties in some areas’ (NITC).

Public perceptions and communication are linked, such as issues related to technology and its capacity,

I think what people don’t necessarily know or understand is the enormous amount of progress we’ve made from a technology perspective in this space. I do think that needs to be really clear. We’re not working off spreadsheets anymore. That’s where we started. We now have a national system that everybody signs up to, that is integrated into the National Health Index and other databases, that is a clear connection to a number of things. So, we’re not working in a technology vacuum (NITC).

And issues related to definitions,

The public, they don’t really understand what the difference is between a close and a casual contact (NITC).

The PHU staff also commented on this public understanding of the technology issue,

I don’t think that is widely known that is how that works. Because I’ve actually been on the door of a place as people coming in asking them to sign in with the App. We will get a lot of people say no because they think—when you talk to them they think information is going direct to the government and that it can be accessed at any time (PHU).

There was some frustration about the lack of understanding from the public on what contact tracing involves, or at the very least highlighting the importance of understanding current perceptions and expectations and communicating carefully and consistently.

As government employees, the focus group participants were very aware of who they report to and the subsequent pressures,

It’s the political pressure and the constant ‘more information, more information’ requirements and having this perception that that adds value and actually will keep us safer (NITC).

Similarly, how the information is communicated was widely commented on,

All of us from a caller in a call centre, to a politician, to us here, everybody and the public need to share an understanding of some key things. The more layers of complexity we put in, the harder it is to achieve the overall result of stopping transmission (NITC).

There was unanimous agreement that communication needs to be simple and consistent, ‘If you have that confusion there’s chaos. We’re worried about chaos. We need simplicity’ (NITC). Similarly, a PHU participant noted, there are ‘still a lot of naysayers out there’, therefore there needs to be a lot of communication on this, ‘to get people to buy into it’ (PHU).

### Operational model

The operational model covers the processes and mechanisms for contact tracing. There were concerns or considerations about the process itself, data (sovereignty), workload and ease of use.

I think it’s just such a change in the operating model. I think that this all needs thought. I don’t think that we’ve thought out all of these pathways and potential ways people want to communicate with us (NITC).

Not 100% sure how the benefits will be better than what we do now… how are we going to operationalise it (PHU)?

Considering the Card, it was noted ‘we have to be very conscious about what the unintended consequences are of these tools?’ (NITC) and an awareness that technology is only part of the toolbox,

There’s always going to be a need to have a conversation with the close contacts. I don’t think we will ever get away from that. Even if we are able to get a notification to them we will still need to follow up with the phone call (NITC).

Keep in mind what our game is, 80% in 48 hours. If we get 80% that’s good enough cause it’s a game of speed. … If we miss 20% that’s fine because we’ll still be below the r value. That’s our target, we get 80, we’re below 1, we’re winning. Spending all this extra time to get to up to 90 might actually slow us down (NITC).

A key concern or consideration from the focus group participants was efficacy of the operational model, that is, the whole framework in which contact tracing is operationalized. Regarding new technologies, all focus groups said that any changes needed to have evidence that efficacy would be improved across the model. Participants were not negative about possible changes, ‘If there were ways we could get through [using new technology] I think it really could be of a huge benefit’ (NITC) and it could be ‘helpful to get close contacts’ (PHU). But in a note of warning,

It means quite a change to the operation model. We’ll have to start considering things differently at a public health as well as a centralised level. Because we will not get rid of the interview, the human contact is going to be always our primary source. But we are now going to have different sources that are going to give us additional or different information. However, the information is normally we say, people know who their close contacts are. With the App and QR codes and the Bluetooth, we won’t know that. So, you lose that connection between the two, …, it will change how we do contact tracing (NITC).

The NITC group noted the ‘art’ of contact tracing, where PHU staff have to investigate, follow up, talk with people, not in an impersonal clinical way, but rather working with the individual and whānau (family), helping them to understand what is going on.

As I was saying about the art, there is always going to be this human element to it. Because someone has to assess all the data and see what’s useful and what’s not. It’s kind of about, can the technology actually make the traditional contact tracing process better (NITC).

You can’t programme contact tracing case investigation. Cause if you think about it from a scientific perspective you don’t have most of the things under control. You are trying to direct fast decisions about what to do to manage the new diseases, some you can’t see (NITC).

The ‘art’ comment relates to the data volume issue, as a NITC participant said, ‘There’s a lot of interpretation of information and I think that’s where … having more information may not help do that’ (NITC).

If a new model that includes DCT is to be instigated, it needs to be as clear and simple as the current model, and verifiably more efficacious. However, if in this ‘new operational model’ there is a lack of personal connection, because of an electronic alert to call Healthline, participants question its efficacy. What was not clear or understood by the focus group participants was how a new model would integrate the many contact tracing mechanisms, from personal contact to digital notification consistently. The operational model was further commented on regarding the complexity of digital notification, highlighting the concerns around a de-centralized operating model for these data; one where case investigators have no control over the number of notifications sent or whom they are sent to. There is no way to correlate that with what has been found in case investigations. Data integrity was highlighted as a possible issue with further technology,

We don’t necessarily know if there are duplicates coming through and if everything’s automatically… potentially going to be coming through the system. I suppose that scared me a little bit (NITC).

Uncertainties about data integrity is further linked to public trust. That is, if ‘the Ministry’ gets it wrong, erosion of public trust and future compliance is likely. Focus group participants discussed the hard-to-reach contacts, acknowledging this was a data issue, but also potentially an equity issue, given some may be Māori, Pacific or marginalized communities. This concern was noted by an NITC focus group member,

One of the biggest problems is the few—but the people that we try and contact multiple times. [We] try and find other information for them multiple times and we still don’t get in touch with them. It remains a concern for me, I think if they are going to become a case then we would know about it. … There’s always a burning ember somewhere.

Workload issues were also raised by a number of participants, with questions about whether something like the Card would increase the data available, but not necessarily the efficacy of contact tracing. That is, considering the trade-off between data management, time and effective contact tracing, as NITC participants noted,

That means that if we had to talk to all of them [potential close contacts identified by the Card], some process that can’t manage volume efficiently, then that could slow everything down and speed is everything (NITC).

The main concerns I have around it are the capacity to respond to the work that it generates so it relates a bit to the operating model and thinking about the best use of finite resources (NITC).

Similarly, the PHU staff questioned the cost-effectiveness of new technology in relation to the workload and ‘resource intensive issues’.

Workload concerns extended beyond traditional contact tracing work to those related to reporting to media, ministers and the public. If the data were available, there were concerns about doing endless information requests,

So, we would do a push notification to say you have symptoms go and have a test, so the next thing will be that we need to monitor that.

It’s true, no I’m not being cynical I’m being realistic. That’s the problem. I’ll come back to that. So, if we had that option which in itself would be very doable. We’ll do a push notification, we give people advice, then the thing will be, well how do you know they did it [got tested]?

How many people of those casual contacts have been tested? How many have had the results back and who are those people?

The operational model efficacy issue is also related to public perceptions, with a call to keep it simple and easily communicated, ‘It’s necessary to promote the benefits’ (NITC). Another efficacy issue was the lack of interaction (exposure notification) with the public, resulting in decreased enthusiasm for using the App. That is, ‘all the work they [the public] do to keep track of their movements doesn’t result in anything for them’ (NITC) because there are few outbreaks, therefore few notifications. This may be the same for a Bluetooth-based approach.

Who controls, owns and manages the data was also a consideration for the focus groups. Te Arawa were acknowledged by the NITC group as ‘leading a lot of the work’ in relation to the Rotorua trial. This raises issues related to iwi-led work, levels of control of information collected, and the process, should a COVID case be found.

There are clearly operational matters to clarify,

That’s what worries me about all this. I don’t know what the data is between a case interview and knowing and understanding who the close contacts are vs what goes out and is picked up by the Card or Bluetooth App (NITC).

The fact is that quarantine is an intervention with consequences, they are real and potential harms that people suffer personally, from quarantine. The accuracy is really important, I think you’re right to confirm with them their quarantine stage cause that’s… I don’t think we could have a scenario where people are put in quarantine that we never follow up on. I can imagine it but I think it would be difficult to manage (NITC).

The PHU participants discussed the risk assessment processes, suggesting it can be variable, geographically dependent and related to the nature of how ‘close contact’ is defined and evolving. This ‘evolution’ included a ‘casual plus’ definition, which was between a casual and close contact. They suggested households were the biggest risk, with public setting contacts often less well remembered. While Bluetooth technology could help to mitigate this risk, there was a clear message that the ‘interviews are really important’ for contact tracing, so like other participants, any technology would be additional to existing approaches.

## DISCUSSION

Findings suggest that contact tracers and policy makers appreciate that digital technology could help with contact tracing, with the acknowledgement that countries like Taiwan had avoided lockdowns with strict COVID-19 response measures, including DCT technology. However, for many participants it was seen only as a supplement to existing contact tracing systems. Major concerns around the equitable application of DCT, the adopted operational model and the potential adverse impacts remained. Specifically, concerns around citizen burden of isolating, mixed messages to public and workload of both case investigators and civil servants who were already at capacity.

Participants shared many of the concerns about DCT solutions as the general public, policy makers and researchers (Altmann *et al.*, 2020; Colmar Brunton, 2020; Von Wyl *et al.*, 2020). Equity concerns were a central theme for participants, which are also reflected in international policy documents on DCT (World Health Organization, 2020) and reviews on digital services in general (Foley *et al.*, 2020). However, while not directly asked, participants did not comment on the equity implications of the current model, that is, the close contact tracing success rate by case and per close contact is much higher for New Zealand European than for Māori (Ministry of Health, 2020b). When 80% within 48 h is the key performance metric, we need to understand the 20% of untraced contacts are unlikely to be random and how this may exacerbate existing inequities. Privacy was another major concern for participants, which has been covered extensively by existing research (Von Wyl *et al.*, 2020; World Health Organization, 2020). However, participants were also concerned how these privacy concerns were perceived by the public and the impact it may have on the social licence and efficacy of other public health interventions (e.g. isolation). That is, perceived or real privacy breaches may undermine their current contact tracing activities and the wider COVID-19 response.

Communication and public perception were an important theme. Participants were frustrated by the lack of public awareness and communication about the contact tracing process and overemphasis on DCT solutions. A major contributing factor to this frustration is the scarcity of research and public discourse utilizing the expert opinion of end-users. Another concern was the major public misunderstanding about how these devices actually worked (Williams *et al.*, 2021). Misinformation and disinformation about these solutions can create increasing public resentment towards the entire COVID-19 response or feed existing conspiracy beliefs (Romer and Jamieson, 2020).

Participants raised concerns over increased workload and the model used to operationalize the DCT data. There was wide agreement that if implemented, the technology would not replace any of the core case investigation processes related to close contacts. In fact, there was consensus that there should be minimal or no application of the data to identify or notify close contacts. Instead, end-users saw value in the identification of casual contacts. Additionally, there was a perceived value in the technology providing support to response measures adjacent, but not directly related to, contact tracing such as prioritized testing and source identification.

### Limitations

The study provides insights into the operational limitations of DCT through the expert opinion of end-users, contact tracers and contact tracing professionals. One limitation is that our research occurred at one point in time and focussed on an issue undergoing rapid development. It is possible some of the views expressed by participants could already have changed given advancements and empirical data on DCT solutions. For example, New Zealand integrated the Google/Apple Bluetooth Exposure Notification Framework around 6 months after our first focus group (10 December 2020). However, the high-level issues raised by participants are likely to be relevant through and beyond the pandemic for the implementation of technologies in other public health interventions. Another limitation was we were focussed on two possible Bluetooth solutions (the Card or phone App) that do not represent the entire suite of DCT solutions. It is likely participants would have stronger or softer perceptions towards alternative technologies (eg, GPS surveillance). The participants also noted briefly other forms of DCT already in use such as electronic bank details and transport cards, but noted these were only available at the digression of the case or contact. It is unclear if participants would support access to these records without the case’s explicit consent. Lastly, few participants were Māori; reflecting the wider Māori contact tracing capacity in New Zealand. The lack of Māori contact tracers represents a major limitation of New Zealand’s contact tracing system and likely reduces its efficacy in Māori communities, further exacerbating existing inequities. Of particular concern, is the lack of Māori in policy and governance positions (eg, NITC) granted the power to make system-wide decisions. It is to be hoped that establishment of the Māori Health Authority as part of the recently announced health services reform in New Zealand may go some way to mitigating such discrepancies (Health and Disability Review Transition Unit, 2021).

## CONCLUSIONS

Ultimately, end-users said there was a place for DCT technologies in the response to COVID-19 and future pandemics. To be effective, DCT technologies require strong privacy safeguards, a clear and simple communication strategy, interoperability with the existing contact tracing system, and a robust foundation of health equity.
